# Tryptanthrin Down-Regulates Oncostatin M by Targeting GM-CSF-Mediated PI3K-AKT-NF-κB Axis

**DOI:** 10.3390/nu16234109

**Published:** 2024-11-28

**Authors:** Na-Ra Han, Hi-Joon Park, Seong-Gyu Ko, Phil-Dong Moon

**Affiliations:** 1 College of Korean Medicine, Kyung Hee University, Seoul 02447, Republic of Korea; nrhan@khu.ac.kr; 2 Korean Medicine-Based Drug Repositioning Cancer Research Center, College of Korean Medicine, Kyung Hee University, Seoul 02447, Republic of Korea; epiko@khu.ac.kr; 3 Department of Anatomy & Information Sciences, College of Korean Medicine, Kyung Hee University, Seoul 02447, Republic of Korea; acufind@khu.ac.kr; 4 Department of Preventive Medicine, College of Korean Medicine, Kyung Hee University, Seoul 02447, Republic of Korea; 5 Center for Converging Humanities, Kyung Hee University, Seoul 02447, Republic of Korea

**Keywords:** tryptanthrin, oncostatin M, neutrophils, inflammation

## Abstract

Background: Oncostatin M (OSM) is involved in several inflammatory responses. Tryptanthrin (TRYP), as a natural alkaloid, is a bioactive compound derived from indigo plants. Objectives/ Methods: The purpose of this study is to investigate the potential inhibitory activity of TRYP on OSM release from neutrophils using neutrophils-like differentiated (d)HL-60 cells and neutrophils from mouse bone marrow. Results: The results showed that TRYP reduced the production and mRNA expression levels of OSM in the granulocyte–macrophage colony-stimulating factor (GM-CSF)-stimulated neutrophils-like dHL-60 cells. In addition, TRYP decreased the OSM production levels in the GM-CSF-stimulated neutrophils from mouse bone marrow. TRYP inhibited the phosphorylation of phosphatidylinositol 3-kinase (PI3K), AKT, and nuclear factor (NF)-κB in the GM-CSF-stimulated neutrophils-like dHL-60 cells. Conclusions: Therefore, these results reveal for the first time that TRYP inhibits OSM release via the down-regulation of PI3K-AKT-NF-κB axis from neutrophils, presenting its potential as a therapeutic agent for inflammatory responses.

## 1. Introduction

Inflammation is an immune reaction that results from a wide range of factors [1]. Acute inflammation is caused by infection or exposure to substances and is characterized by pain, redness, swelling, and heat. However, chronic inflammation can lead to serious and potentially life-threatening conditions [2,3]. Inflammation is also closely related to the development and malignant progression of a variety of cancers [4,5]. Oncostatin M (OSM) is well known to be a growth inhibitor of several types of tumor cells [6]. However, OSM is an inflammatory cytokine and implicated in the pathogenesis of inflammatory responses [7,8]. OSM has thus been identified as a therapeutic target for inflammatory responses as well as several types of cancers [9].

OSM is produced by neutrophils, osteoblasts, or bone marrow macrophages [7]. Neutrophils, as an important component of the innate immune response, are the first line of defense against pathogens arriving at sites of acute inflammation [10]. Neutrophils also contribute to the activation of other immune cells in sites of chronic inflammation [11]. Granulocyte macrophage–colony-stimulating factor (GM-CSF) functions as a differentiation factor for neutrophil precursors; stimulates mature neutrophils; and affects phagocytosis, degranulation, or transmigration of neutrophils [12]. GM-CSF leads to the release of OSM from neutrophil granules and augments inflammatory responses [12]. GM-CSF-induced OSM expression is regulated via the phosphatidylinositol 3-kinase (PI3K), AKT, and nuclear factor (NF)-κB signaling pathway in neutrophils [13].

Human promyelocytic leukemia (HL-60) is a commonly used surrogate cell line model of neutrophils [14]. Treatment of HL-60 cells with dimethyl sulfoxide (DMSO) induces differentiation into neutrophil-like cells [15,16]. The DMSO-induced neutrophils-like differentiated (d) HL-60 cells have already been applied in studies to elucidate neutrophil functions [15,17]. The bone marrow of mice serves as a reservoir for isolating large numbers of neutrophils [18]. The bone marrow-derived neutrophils have also been used in neutrophil research [19,20].

Tryptanthrin (TRYP), as a natural alkaloid, is a bioactive compound derived from medicinal indigo plants [21,22,23]. TRYP has been shown to exert anti-cancer [24], anti-atherosclerosis [25], anti-atopic dermatitis [26], and anti-neuroinflammatory activities [27]. Regarding neutrophils, there are reports that TRYP exerts pharmacological effects by regulating leukotriene formation in calcium ionophore or lipopolysaccharides-stimulated neutrophils [28,29]. However, no studies have investigated the regulatory effect of TRYP on OSM in GM-CSF-stimulated neutrophils. Considering the pharmacological effects of TRYP mentioned above, we hypothesized that TRYP may regulate OSM levels in the GM-CSF-stimulated neutrophils. Here, we demonstrate the effectiveness of TRYP on OSM in neutrophils-like dHL-60 cells and neutrophils from mouse bone marrow, and we explore mechanisms underlying these effects.

## 2. Materials and Methods

### 2.1. TRYP

TRYP (purity ≥ 99%) was bought from Sigma-Aldrich (St. Louis, MO, USA). It was dissolved in DMSO and then diluted with culture media. 

### 2.2. Cells

HL-60 cells (Korean Cell Line Bank, Seoul, Republic of Korea) were differentiated into neutrophil-like dHL-60 cells by treating them with 1.3% *v*/*v* DMSO for 7 days. Bone marrow-derived neutrophils were isolated using the density gradient method according to an established protocol [18,30], which was approved by the Animal Care Committee (#KHSASP-24-623). Briefly, bone marrow from male C57BL/6 mice was extracted by flushing with RPMI 1640 (Gibco, Waltham, MA, USA). Neutrophils were enriched by density centrifugation using Histopaque 1077 (Sigma-Aldrich, Cat. No. 10771) and Histopaque 1119 (Sigma-Aldrich, Cat. No. 11191). Neutrophils were collected from the interphase of the two Histopaque layers. Both HL-60 cells and bone marrow-derived neutrophils were cultured in RPMI 1640 with 10% heat-inactivated fetal bovine serum (Merck Millipore, Burlington, MA, USA) and 1% penicillin/streptomycin antibiotics (Gibco) at 37 °C in 5% CO_2_ and 95% air. 

### 2.3. Cell Viability Assay 

The cell viability was analyzed using a 3-(4,5-dimethylthiazol-2-yl)-2,5-diphenyltetrazolium bromide (MTT) assay after cells were incubated with TRYP for 1 h and then with recombinant GM-CSF (5 ng/mL, Cat. No. 215-GM (for HL-60 cells, *E. coli*-derived human GM-CSF protein Ala18-Glu144), Cat. No. 415-ML (for mouse neutrophils, *E. coli*-derived mouse GM-CSF protein Ala18-Lys141, with an N-terminal Met), R&D Systems, Minneapolis, MN, USA) for an additional 4 h. 

## 3. Results

### 3.1. Effect of TRYP on OSM Release 

**Figure 1:**
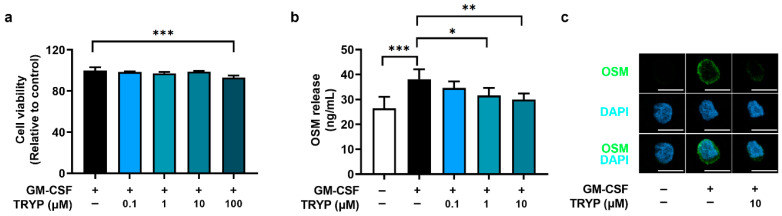
TRYP reduces OSM release. dHL-60 cells were stimulated with GM-CSF, with or without TRYP, for 4 h. (**a**) The cell viability was assessed using an MTT assay. (**b**) OSM production was examined using ELISA. (**c**) Representative images for OSM were obtained by confocal microscopy (scale bar, 10 µm). * *p* < 0.05; ** *p* < 0.01; *** *p* < 0.001.

**Figure 2:**
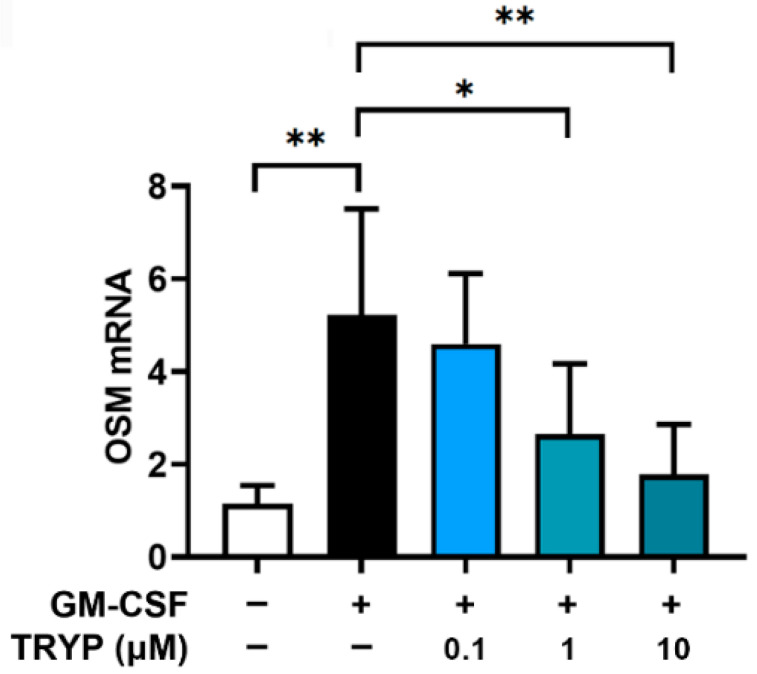
TRYP reduces the OSM mRNA levels. dHL-60 cells were stimulated with GM-CSF, with or without TRYP, for 30 min. OSM mRNA expression was assessed with qPCR. * *p* < 0.05; ** *p* < 0.01.

The cell viability was first investigated at different concentrations of TRYP in GM-CSF-stimulated neutrophil-like dHL-60 cells using an MTT assay. Cells were incubated with TRYP for 1 h and then with recombinant GM-CSF for 4 h. The cell viability was suppressed at a concentration of 100 µM of TRYP (Figure 1a). The dose–response curve for cell viability clearly shows the concentration-dependent effects of TRYP on cell viability (Supplementary Figure S1). In subsequent experiments, we used TRYP at and below 10 μM less than 100 µM [26,31]. Additionally, we confirmed that TRYP (0.1 µM–10 µM) did not affect the survival rate at 12 h and 24 h after GM-CSF stimulation (Supplementary Figure S2). We next assessed the effects of TRYP on OSM release in the GM-CSF-stimulated neutrophil-like dHL-60 cells. Consistent with previous reports [12,13], the GM-CSF treatment augmented the OSM release from neutrophil-like dHL-60 cells. However, the treatment with TRYP dose-dependently reduced the OSM release compared to the GM-CSF-stimulated control group (Figure 1b). The dose–response curve for OSM release clearly demonstrates a concentration-dependent inhibitory effect of TRYP on OSM release (Supplementary Figure S3). The decreased expression levels of OSM by TRYP were visualized by immunofluorescence microscopy (Figure 1c). Furthermore, GM-CSF treatment resulted in a progressive release of OSM from bone marrow-derived neutrophils, with peak induction at 4 h (Supplementary Figure S4a). The treatment with TRYP dose-dependently reduced the OSM release from bone marrow-derived neutrophils (Supplementary Figure S4b), with no effect on neutrophil viability (Supplementary Figure S4c). In addition, Figure 2 shows that the GM-CSF-stimulation significantly increased mRNA expression of OSM. This effect caused by GM-CSF was clearly reversed by TRYP treatment.

### 3.2. Effect of TRYP on Phosphorylation of PI3K 

**Figure 3:**
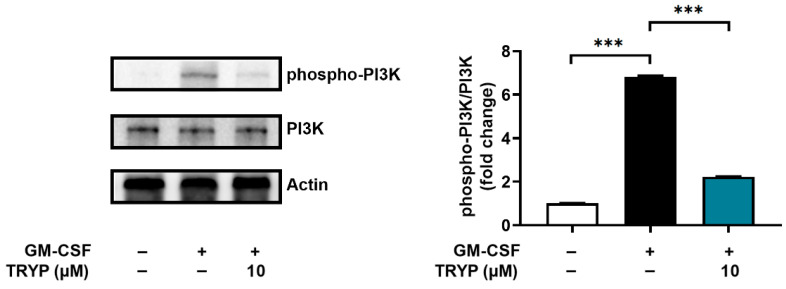
TRYP reduces the phosphorylation of PI3K. The phospho-PI3K levels were analyzed using immunoblots. Quantitative analysis of blots from three independent experiments is displayed in the right panel. *** *p* < 0.001.

A PI3K inhibitor reduced the OSM expression in several cells, including neutrophils [13,32]. In addition, the authors’ previous report revealed that wortmannin, a PI3K inhibitor, reduces the GM-CSF-induced OSM levels in neutrophil-like dHL-60 cells [13]. We thus performed Western blot analysis on the phosphorylation of PI3K in neutrophil-like dHL-60 cells to further elucidate the underlying mechanisms driving the regulatory effect of TRYP on GM-CSF-induced OSM production. As expected, TRYP significantly suppressed the phosphorylation levels of PI3K increased by GM-CSF (Figure 3).

### 3.3. Effect of TRYP on Phosphorylation of AKT 

**Figure 4:**
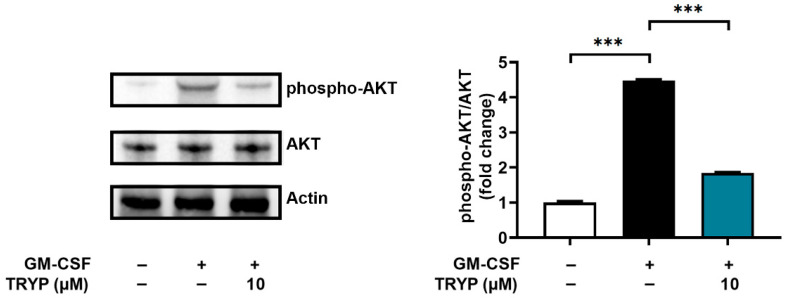
TRYP reduces the phosphorylation of AKT. The phospho-AKT levels were measured by WB analysis. Quantitative analysis of blots from three independent experiments is displayed in the right panel. *** *p* < 0.001.

AKT, as a key downstream target of PI3K, is a critical component of signaling following PI3K activation [33]. In addition, MK 2206, an AKT inhibitor, decreased the GM-CSF-induced OSM levels in neutrophil-like dHL-60 cells [13]. WB analysis demonstrated that TRYP significantly inhibited the phosphorylation levels of AKT compared to the GM-CSF-stimulated control group (Figure 4).

### 3.4. Effect of TRYP on Phosphorylation of NF-κB

**Figure 5:**
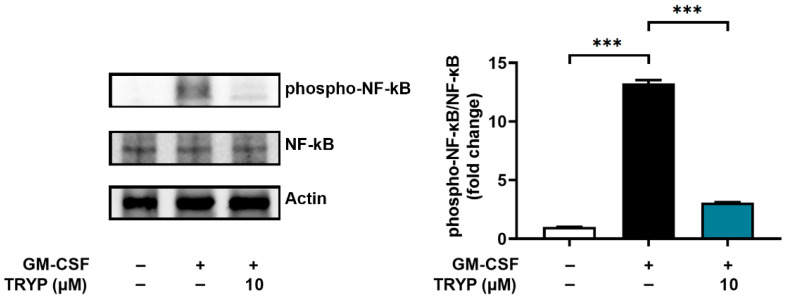
TRYP reduces the phosphorylation of NF-κB. The phospho-NF-κB levels were analyzed using immunoblots. Quantitative analysis of blots from three independent experiments is displayed in the right panel. *** *p* < 0.001.

AKT induces phosphorylation of NF-κB and thus modulates the transcriptional activity of NF-κB [33]. PDTC, a NF-κB inhibitor, suppressed the GM-CSF-induced OSM levels in neutrophil-like dHL-60 cells [13]. Subsequently, we examined whether TRYP would regulate the phosphorylation of NF-κB. TRYP significantly inhibited the phospho-NF-κB levels increased by GM-CSF (Figure 5). These results (Figure 3, Figure 4 and Figure 5) indicated that the anti-inflammatory effect of TRYP resulted from the inhibition of OSM levels regulated by PI3K-AKT-NF-κB signaling pathways in neutrophils.

## 4. Discussion

Medicinal plants are composed of bioactive compounds or secondary metabolites that possess biological activity [34]. Given the various side effects and drug resistance, medicinal plants may be a useful alternative treatment [34,35]. Bioactive compounds or phytonutrients, as health-promoting biologically active compounds found in medicinal plants or plant-based foods, have been actively researched scientifically for human health [36]. Phytonutrients include alkaloids, phenolics, and several other food components [36]. These have a variety of useful properties for human health, such as inhibition of inflammatory and oxidative reactions [37,38]. Indigo is commonly used to dye textile products, but it is also used as a dye in foods and pharmaceuticals [39]. Indigo also contains a significant amount of phytonutrients such as alkaloid, total phenols, tannins, saponins, and flavonoids [40] and has various pharmacological efficacy in the inhibition of oxidation, inflammation, and angiogenesis reactions [23,41]. Alkaloids are used in medicine, particularly for their anesthetic, cardioprotective, cytostatic, and anti-inflammatory effects [42]. TRYP is a natural alkaloid found in medicinal indigo plants [21]. This study revealed a novel pharmacological effect of TRYP by demonstrating that TRYP inhibits OSM production in neutrophils. This is the first work to demonstrate the regulatory effect of TRYP on neutrophil-derived OSM. However, additional experiments are needed to verify the effectiveness of TRYP against OSM in several inflammatory models.

Inflammation is a complex cellular process that involves immune cells and signaling molecules [43]. Several immune-related factors are involved in regulating both inflammation and cancer progression [44]. OSM, as a cytokine fulfilling several functions, is most closely associated with leukemia inhibitory factors [6]. However, a pro-tumorigenic role of OSM has also been reported in breast cancer or pancreatic cancer [45]. Interestingly, OSM in inflammatory responses plays opposing roles, suggesting that it may vary depending on the stage of inflammation and the inflammatory environment. The anti-inflammatory properties of OSM were established in experimental models of rheumatoid arthritis and lung inflammation [46,47]. In contrast, there is increasing evidence that OSM has pro-inflammatory properties [6,48,49]. Retrospective studies conducted in Hong Kong and Atlanta found that increased OSM was correlated with disease severity in COVID-19 infection [50]. OSM expression was increased in inflamed tissue from patients with Crohn’s disease and ulcerative colitis compared to non-inflamed colonic lesions of these patients [51]. OSM expression was also increased in inflamed tissues of patients with inflammatory bowel disease, which was closely related to disease severity [52]. Subcutaneous injection of OSM induced an acute inflammatory response in mice [48]. There are several reports suggesting the possibility that TRYP’s effects on OSM may influence current treatments for inflammatory diseases. OSM knockout mice with inflammatory bowel disease had reduced severity of overall pathology and disease features compared to wild-type mice [52]. The administration of anti-OSM antibodies to mouse models with arthritis significantly improved the arthritis severity [53]. In addition, a recent study has shown that a bioactive compound, berberine, attenuates chronic ulcerative colitis by inhibiting OSM production [54]. We demonstrated that OSM production was increased in the GM-CSF-stimulated neutrophil-like dHL-60 cells and neutrophils from mouse bone marrow, and TRYP decreased the increased OSM production, suggesting an anti-inflammatory effect of TRYP and its potential applications in various OSM-mediated inflammatory responses in this study. Nevertheless, further studies are warranted to study the efficacy of TRYP on the clinical significance of OSM in several OSM-mediated inflammatory models.

The AKT which functions as a critical downstream target of PI3K, and PI3K phosphorylate numerous protein targets that regulate several cellular processes [33,55]. In addition, the PI3K-AKT pathway is part of a signaling pathway required to induce key immune and inflammatory responses because PI3K-AKT functions as upstream kinases for NF-κB activation [56,57]. The NF-κB transcription factor is a critical mediator of the inflammatory responses, and several studies have demonstrated that NF-κB plays an important role in linking inflammation and cancer [58]. OSM has been reported to be expressed through the PI3K-AKT-NF-κB signaling pathway in osteoblasts [32]. In our previous report, we revealed that OSM expression was regulated via the PI3K-AKT-NF-κB signaling pathway in neutrophils [13]. Binding of GM-CSF to its receptor activates src-tyrosine kinase Lyn (LYN), which activate PI3K-AKT signaling in human neutrophils [59]. The serine/threonine protein kinase pim (PIM) and hematopoietic cell kinase (HCK, tyrosine protein kinase) interact with PI3K-AKT and induce PI3K-AKT phosphorylation [60,61,62]. Han et al. [63] indicated that indigo and TRYP potentially bind LYN, PIM1, and HCK and inhibit the expression of these proteins. We found that TRYP inhibits OSM levels via the PI3K-AKT-NF-κB signaling pathway in this study. Thus, we suggest that TRYP might down-regulate PI3K-AKT-NF-κB signaling by interacting with these kinase proteins. However, further studies are needed to elucidate the exact molecular mechanisms by analyzing the direct binding of TRYP to these proteins in GM-CSF-stimulated neutrophils. 

## 5. Conclusions

Collectively, this study is the first to demonstrate that TRYP presented a significantly beneficial effect on neutrophils by regulating the OSM production through the PI3K, AKT, and NF-κB pathways. Therefore, TRYP may have the potential to treat OSM-mediated inflammatory diseases. However, this study has a limitation in that no in vivo experiments were conducted as part of the study. This study focused on the in vitro effects of TRYP on the OSM release from neutrophils. However, the findings of this study may provide foundational data and persuasive evidence to support the exploration of TRYP in future studies of animal models of OSM-mediated inflammatory responses. It also provides insight into the potential utility of TRYP in addressing OSM-mediated inflammatory diseases. However, in vivo studies clarifying functional OSM in several inflammation models are needed to demonstrate the anti-inflammatory effects of TRYP.

## Data Availability

The data sets used and/or analyzed during the current study are available from the corresponding author due to legal reasons.

## Supplementary Materials

The following supporting information can be downloaded at: https://www.mdpi.com/article/10.3390/nu16234109/s1, Figure S1: Dose-response curve for cell viability of TRYP; Figure S2: The effect of TRYP on survival rate; Figure S3: Dose-response curve for OSM release of TRYP; Figure S4: Effect of TRYP on OSM release from bone marrow-derived neutrophils. Table S1. PCR primer sequence.
