# Supplementary material for: Tryptanthrin Down-Regulates Oncostatin M by Targeting GM-CSF-Mediated PI3K-AKT-NF-κB Axis
Source: Nutrients. 2024 Nov 28;16(23):4109. doi: 10.3390/nu16234109 (PMC11643981; doi:10.3390/nu16234109)
Supplement: Supplementary file 1 [file nutrients-16-04109-s001.zip › nutrients-3274692-supplementary.pdf]

Tryptanthrin Down-Regulates Oncostatin M by Targeting GM-CSF-Mediated PI3K-AKT-NF-κB Axis

Na-Ra Han [1,2], Hi-Joon Park [3], Seong-Gyu Ko [2,4] and Phil-Dong Moon [5,*]

[1] College of Korean Medicine, Kyung Hee University, Seoul 02447, Republic of Korea; nrhan@khu.ac.kr
[2] Korean Medicine-Based Drug Repositioning Cancer Research Center, College of Korean Medicine, Kyung Hee University, Seoul 02447, Republic of Korea; epiko@khu.ac.kr
[3] Department of Anatomy & Information Sciences, College of Korean Medicine, Kyung Hee University, Seoul 02447, Republic of Korea; acufind@khu.ac.kr
[4] Department of Preventive Medicine, College of Korean Medicine, Kyung Hee University, Seoul 02447, Republic of Korea
[5] Center for Converging Humanities, Kyung Hee University, Seoul 02447, Republic of Korea
* Correspondence: pdmoon@khu.ac.kr

Materials and Methods

OSM Assay

dHL-60 cells and bone marrow-derived neutrophils were incubated with TRYP for 1 h and then with recombinant GM-CSF for an additional 4 h. OSM production was determined using a sandwich ELISA method (R&D systems).

Immunofluorescent Staining

For OSM expression analysis, cells were incubated with TRYP for 1 h and then with recombinant GM-CSF for an additional 4 h. Immunofluorescent staining was conducted with anti-OSM (R&D systems) and anti-goat IgG H&L (Alexa Fluor 488, Abcam). Images were analyzed through the utilization of Zeiss LSM800 fluorescence microscope (Carl Zeiss, Oberkochen, Germany).

qPCR

Cells were incubated with TRYP for 1 h and then with recombinant GM-CSF for an additional 30 min. Total RNA was extracted using the easy-BLUE™ Total RNA Extraction Kit (iNtRON Biotech Inc., Republic of Korea). cDNA synthesis from total RNA was performed using a cDNA synthesis kit (Bioneer Corporation, Republic of Korea). Amplification was performed in Real-Time PCR System (Applied Biosystems, Waltham, MA, USA) using Power SYBR® Green Master Mix (Thermo Fisher Scientific, Waltham, MA, USA) with each primer (Supplementary Table S1). Gene expression levels were determined after normalization to GAPDH. The relative levels of mRNA were measured using comparative threshold cycle analysis.

Supplementary Table S1. PCR primer sequence

**Target gene**		**Sequence** 5′ - 3′
NM_020530	OSM F	GCTCACACAGAGGACGCTG
	OSM R	GGAGCACGCGGTACTCTTTC
NM_002046	GAPDH F	TCGACAGTCAGCCGCATCTTCTTT
	GAPDH R	ACCAAATCCGTTGACTCCGACCTT

Western Blot Analysis

Cells were incubated with TRYP for 1 h and exposed to recombinant GM-CSF for additional times (i.e., PI3K/15 min, AKT/30 min, and NF-κB/30 min). The cell lysates were prepared in accordance with previous protocols [21,26]. Proteins were subjected to electrophoresis using 10% gel containing sodium dodecyl sulfate. The transferred nitrocellulose membranes (Amersham™, Chicago, IL, USA) were incubated with primary antibodies of phosphorylated (phospho)-PI3K p85 (Tyr 467, Cat. No. 17366, 1:1000 dilution, Cell Signaling Technology, Danvers, MA, USA), PI3K p85 (Cat. No. sc-423, 1:500 dilution), phospho-AKT (Ser 473, Cat. No. sc-514032, 1:500 dilution), AKT (Cat. No. sc-81434, 1:500 dilution), phospho-NF-κB p65 (Ser 536, Cat. No. sc-136548, 1:500 dilution), NF-κB p65 (Cat. No. sc-8008, 1:500 dilution), and actin (Cat. No. 8432, 1:500 dilution, Santa

Cruz Biotechnology, Santa Cruz, CA, USA). The membrane was further incubated with corresponding HPR-conjugated secondary antibodies (Santa Cruz Biotechnology). Specific bands were detected with an enhanced chemiluminescence solution (DoGenBio Co., Seoul, Republic of Korea). ImageJ program was used to analyze the bands. The protein levels are expressed as fold change values relative to the un-treated group (blank).

Statistical Analysis

The statistical calculations were performed in Prism 9 (GraphPad Software, Inc., Boston, MA, USA). Results were shown as mean ± standard deviation (SD). Data are representative of three independent experiments performed in duplicate. One-way analysis of variance along with Tukey's *post hoc* test was used to compare multiple groups. Student's unpaired *t*-test was used to analyze a significance between two independent groups (*$p < 0.05$; **$p < 0.01$; ***$p < 0.001$).

Supplementary Figure S1

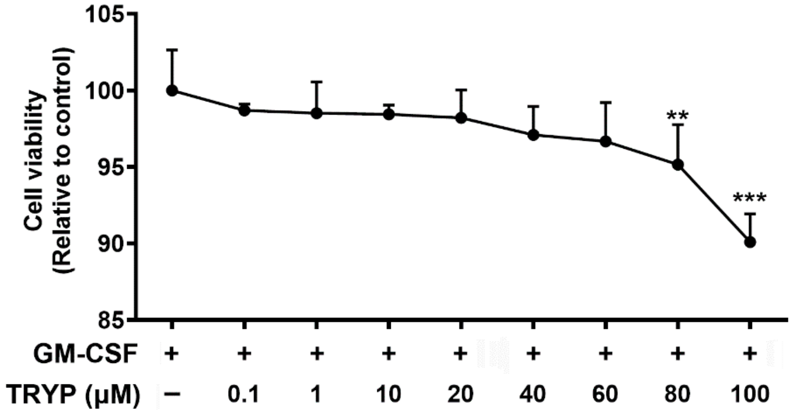

Supplementary Figure S1. Dose-response curve for cell viability of TRYP. dHL-60 cells were incubated with TRYP (0.1 μM – 100 μM) for 1 h and then with recombinant GM-CSF for 4 h. The cell viability was assessed using an MTT assay. $^{**}p < 0.01$ and $^{***}p < 0.001$ vs GM-CSF-treated group.

Supplementary Figure S2

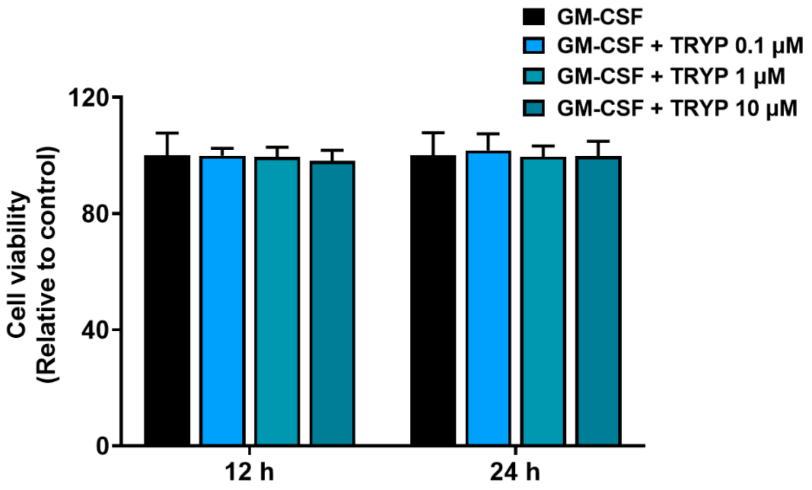

Supplementary Figure S2. The effect of TRYP on survival rate. dHL-60 cells were incubated with TRYP for 1 h and then with recombinant GM-CSF for 12 h and 24 h. The cell viability was assessed using an MTT assay.

Supplementary Figure S3

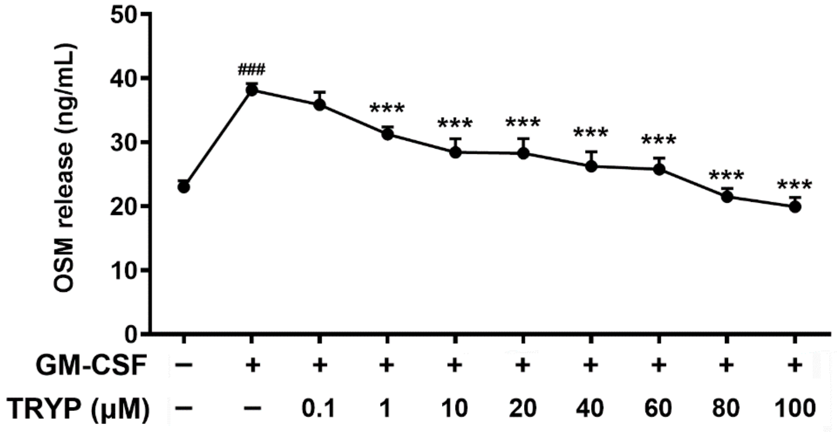

Supplementary Figure S3. Dose-response curve for OSM release of TRYP. dHL-60 cells were incubated with TRYP (0.1 μM – 100 μM) for 1 h and then with recombinant GM-CSF for 4 h. OSM production was examined using ELISA. ###$p < 0.001$ vs blank group, ***$p < 0.001$ vs GM-CSF-treated group.

Supplementary Figure S4

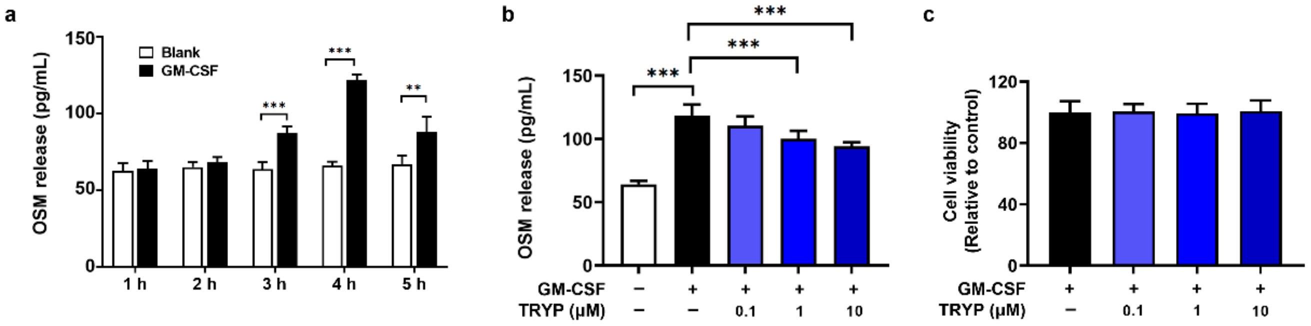

Supplementary Figure S4. Effect of TRYP on OSM release from bone marrow-derived neutrophils. (a) Neutrophils were stimulated with GM-CSF (5 ng/ml) for 1 h - 5 h. Blank, un-treated group. (b) Neutrophils were stimulated with GM-CSF, with or without TRYP for 4 h. OSM production was examined using ELISA. (c) The neutrophil viability was assessed using an MTT assay.
